# Fatigue in children and young people up to 24 months after infection with SARS-CoV-2

**DOI:** 10.1038/s41598-025-24868-x

**Published:** 2025-11-20

**Authors:** Alvin Richards-Belle, Roz Shafran, Natalia K. Rojas, Terence Stephenson, Ewan Carr, Trudie Chalder, Emma Dalrymple, Kelsey McOwat, Ruth Simmons, Snehal M. Pinto Pereira, CLoCk Consortium, Marta Buszewicz, Esther Crawley, Bianca De Stavola, Tamsin Ford, Shruti Garg, Dougal Hargreaves, Anthony Harnden, Isobel Heyman, Shamez N. Ladhani, Michael Levin, Vanessa Poustie, Terry Segal, Malcolm Semple, Kishan Sharma, Olivia Swann, Elizabeth Whittaker

**Affiliations:** 1 Division of Psychiatry, University College London, Maple House, 149 Tottenham Court Road, London, W1T 7NF United Kingdom; 2 UCL Great Ormond Street Institute of Child Health, 30 Guilford Street, London, WC1N 1EH United Kingdom; 3 Division of Surgery & Interventional Science, Faculty of Medical Sciences, University College London, 170 Tottenham Court Road, London, W1T 7HA United Kingdom; 4 Department of Biostatistics & Health Informatics, Institute of Psychiatry, Psychology and Neuroscience, King’s College London, 16 De Crespigny Park, London, SE5 8AF United Kingdom; 5 Department of Psychological Medicine, Institute of Psychiatry, Psychology & Neuroscience, King’s College London, 16 De Crespigny Park, London, SE5 8AF United Kingdom; 6 Immunisations and Vaccine Preventable Diseases, UK Health Security Agency, 61 Colindale Avenue, London, NW9 5EQ United Kingdom; 7 University College London, London, UK; 8 University of Bristol, Bristol, UK; 9 UCL Great Ormond Street Institute of Child Health, London, UK; 10 University of Cambridge, Cambridge, UK; 11 University of Manchester, Manchester, UK; 12 Imperial College London, London, UK; 13 University of Oxford, Oxford, UK; 14 Cambridge and Peterborough NH, Foundation Trust, Cambridge, UK; 15 UK Health Security Agency, London, UK; 16 University of Liverpool, Liverpool, UK; 17 University College London Hospitals NHS Foundation Trust, London, UK; 18 Manchester University NHS Foundation Trust, Manchester, UK; 19 University of Edinburgh, Edinburgh, UK

**Keywords:** SARS-CoV-2, Post-COVID condition, Long COVID, Fatigue, Children and young people, Infectious diseases, Epidemiology, Paediatric research, Fatigue

## Abstract

Persistent fatigue is common following acute SARS-CoV-2 infection. Little is known about post-infection fatigue trajectories in children and young people (CYP). This paper reports on a longitudinal analysis of the Children and Young People with Long COVID study. SARS-CoV-2-positive participants, aged 11-to-17-years at enrolment, responding to follow-ups at 3-, 6-, 12-, and 24-months post-infection were included. Fatigue was assessed via the Chalder Fatigue Scale (CFQ; score range: 0-11, with ≥4 indicating clinical case-ness) and by a single-item (no, mild, severe fatigue). Fatigue was described cross-sectionally and examined longitudinally using linear mixed-effects models. Among 943 SARS-CoV-2-positive participants, 581 (61.6%) met CFQ case-ness at least once during follow-up. A higher proportion of ever-cases (vs. never-cases) were female (77.1% vs. 54.4%), older (mean age 15.0 vs. 13.9 years), and met Post-COVID Condition criteria 3-months post-infection (35.6% vs. 7.2%). The proportion of CFQ cases increased from 35.0% at 3-months to 40.2% at 24-months post-infection; 15.9% meet case-ness at all follow-ups. Single-item mild/severe responses showed sensitivity (≥0.728) and specificity (≥0.755) for CFQ case ascertainment. On average, CFQ scores increased by 0.448 points (95% CI, 0.252 to 0.645) over 24-months, but there were subgroup differences (e.g., fatigue increased faster in females than males and improved slightly in those meeting Post-COVID Condition criteria 3-months post-infection while worsening in those not meeting criteria). Persistent fatigue was prominent in CYP up to 24 months after infection. Subgroup differences in scores and trajectories highlight the need for targeted interventions. Single-item assessment is a practical tool for screening significant severe fatigue.

**Supplementary Information:**

The online version contains supplementary material available at 10.1038/s41598-025-24868-x.

## Introduction

Persistent fatigue has emerged as a common, debilitating symptom following acute SARS-CoV-2 infection^1^. It is the second most common manifestation of Post-COVID Condition (PCC, also known as Long COVID)^2^, and, among children and young people (CYP), the pooled prevalence of fatigue or weakness post-infection is estimated at 16.3% (95% CI, 15.7 to 16.9)^3^. The prevalence among adults with PCC is estimated at 34.8% (95% CI, 17.6 to 57.2)^4^. This differential prevalence highlights the need to investigate post-infection symptoms in CYP separately from adults to ensure their unique health needs are understood and met^2^. This is especially relevant for fatigue since adolescence is a time during which fatigue increases^5^.

A recent systematic review and meta-analysis of 50 controlled studies, which included over 14 million people, reported increased risks of up to 42 symptoms following SARS-CoV-2 infection, but only 13 of these studies were specifically in CYP^6^. A substantial amount of information about paediatric PCC has been provided by the Children and Young People with Long COVID (CLoCk) study, a longitudinal cohort of over 30,000 CYP in England matched on SARS-CoV-2 test result and demographic factors at study invitation^7^. A key aim of CLoCk was to describe the clinical phenotype and prevalence of symptoms following acute infection. To date, follow-ups have been completed at 3-, 6-, 12-, and 24-months post-SARS-CoV-2 testing. Previous CLoCk studies showed that over 24-months, most participants who met the definition for PCC at 3-months post-infection went on to recover. However, 7% continued to meet the definition at all follow-ups^8^. Fatigue, as identified by single-item symptom assessment, was, again, a commonly reported symptom amongst those with persistent PCC^8^.

Despite these advances in our understanding of PCC in CYP, little remains known about fatigue change over time following SARS-CoV-2 infection. For example, does the natural course of post-infection fatigue improve, remain constant, or follow a waxing and waning trajectory, and, do fatigue trajectories vary by demographic, pre-pandemic health, school, and acute infection factors? Such evidence is needed to inform interventions and service delivery, as well as for future pandemic preparedness. To date, much of the knowledge about post-infection fatigue has been derived from single-item assessments of tiredness^1,8–10^, and/or by examining fatigue using validated scales at single time-points^11^. While valuable, both approaches limit in-depth understanding of fatigue trajectories. To fill the above identified evidence gaps, we aimed to investigate reported experiences of fatigue, assessed using a valid and reliable scale^12,13^, among CYP up to 24-months after confirmed SARS-CoV-2 infection. Our specific research questions were:

1. What are the profiles of fatigue at 3-, 6-, 12-, and 24-months post-infection?
2. Is single-item assessment a valid tool for detecting potentially severe fatigue?
3. Do experiences of fatigue change over 24-months post-infection, and do trajectories vary by demographic, pre-pandemic health, school, and acute infection factors?

## Methods

### Participants

The CLoCk study recruited 31,012 CYP in England aged 11-to-17-years at study invitation and matched on SARS-CoV-2 test result according to month of testing (between September 2020 and March 2021), sex at birth, age, and geographic area. Study design is described in detail elsewhere^7,14^. In brief, potential participants were contacted with study information via post by Public Health England (now UK Health Security Agency). Information included a web link for electronic consent and questionnaire completion. SARS-CoV-2 polymerase chain reaction test results were sourced from laboratory information management systems at the UK Health Security Agency, to which reporting by hospitals and laboratories was mandatory during study recruitment.

CLoCk received ethical approval from the Yorkshire and the Humber–South Yorkshire Research Ethics Committee (REC reference: 21/YH/0060). All research was performed in accordance with relevant guidelines/regulations, including the Declaration of Helsinki. All participants provided (electronic) informed consent.

In this analysis, we included CLoCk participants who tested positive for SARS-CoV-2 between January and March 2021 and responded to questionnaires at 3-, 6-, 12-, and 24-months post-testing. This sub-cohort were enrolled 3-months post-testing (i.e., between April and June 2021) and have previously been characterised in Nugawela *et al*^15^. We selected this sub-cohort given definitive ascertainment of SARS-CoV-2 positive status as part of routine national testing, availability of data at four time-points, and completeness of responses over follow-up. We did not include a comparison group of participants testing negative for SARS-CoV-2 at baseline given many of such participants may have been infected during follow-up.

### Measures

At all time-points, participants self-completed questionnaires about their physical and mental health, containing elements of the International Severe Acute Respiratory and emerging Infection Consortium Paediatric COVID-19 follow-up questionnaire^16^. Participants could ask for help completing questionnaires from parents/carers or by contacting the research team. The 3-month post-testing (i.e., at study enrolment in April-June 2021, 3-months after testing in January-March 2021) questionnaire also collected demographics, and retrospective reports of whether participants often felt very tired prior to the pandemic (in early March 2020) and their main symptoms at their SARS-CoV-2 test (between January-March 2021). At all time-points, fatigue was assessed using the Chalder Fatigue Scale (CFQ)^12,13^and single-item assessment^14^. In addition, SARS-CoV-2 testing data were linked to the national Personal Demographic Service by the UK Health Security Agency to provide further data on age at infection, sex at birth, and the 2019 English Index of Multiple Deprivation (IMD, computed at small-area level using participants’ residential postcodes).

#### *Chalder Fatigue Scale (CFQ)*^12,13^

The CFQ is a reliable 11-item scale of fatigue severity designed for use in hospital and community settings, and which has been validated in clinical and non-clinical samples^12,13^. The questionnaire comprises two subscales – physical and mental fatigue. Using a bimodal scoring system, total scale scores range from 0-to-11 and are calculated as the sum of item scores in which four response options of increasing severity (e.g., from ‘less than usual’ to ‘much more than usual’) are assigned values of 0, 0, 1, and 1. Total scores ≥4 indicate ‘case-ness’ (a term which means the score is severe enough to be regarded as a clinical case)^17^. Using a Likert-style scoring system (where item responses options are assigned values of 0, 1, 2, and 3), total scale scores range from 0-to-33, with higher scores indicating greater fatigue severity. The Likert-style scoring system is not typically used to define case-ness; therefore our primary analysis focused on the bimodal system with a supplemental analysis using the Likert-style system^18–20^.

#### Single-item fatigue assessment

All questionnaires contained several single-item assessments of a broad range of symptoms, including “Are you experiencing unusual fatigue/tiredness?” – with three response options (“No”, “Mild fatigue”, “Severe fatigue – I struggle to get out of bed”). This item has not previously been validated, but previous CLoCk cohort publications identified a high prevalence of fatigue according to this item^1,8^, necessitating further exploration of its validity.

### Statistical analysis

We characterised fatigue cross-sectionally at each follow-up using descriptive statistics and longitudinally across all follow-ups using linear mixed-effect models.

#### Descriptive analysis

To characterise profiles of fatigue (research question one) using the standard CFQ bimodal scoring system, we described the total and subscale scores, individual item scores, and the proportions meeting case-ness^12,13^ at each follow-up. We report Cronbach’s α at each follow-up as a measure of reliability.

We compared characteristics of participants that met CFQ case-ness at least once during follow-up (ever-cases) to those who never met case-ness threshold (never-cases). Characteristics included age at infection, sex at birth, ethnicity, IMD quintile, and whether participants reported retrospectively at enrolment: having learning difficulties at school and/or an Education Health and Care Plan (EHCP) before the pandemic (the latter indicating a need for extra learning support in school); often feeling very tired in early March 2020; and unusual fatigue/tiredness as a main symptom at testing in January-March 2021. We compared the proportion that met (vs did not meet) the research definition^21^for PCC 3-months post-infection. As per previous studies^8,15^, this definition was operationalised as (i) experiencing ≥1 symptom from a pre-specified list of 21 symptoms (including an ‘other’ option) and (ii) ‘some’ or ‘a lot of’ problems with mobility, self-care, doing usual activities, having pain/discomfort, or feeling very worried/sad/unhappy as measured using the EuroQol Five Dimensions Youth scale^22^.

To assess the validity of the single-item assessment (research question two), we first explored the relationship between CFQ case-ness and single-item assessments via cross-tabulation. Using CFQ case-ness as a benchmark, we then combined ‘severe’ and ‘mild’ single-item responses (as done in previous studies using CLoCk data^1,8^) and calculated sensitivity, specificity, Youden’s J, positive and negative predictive values at each time-point. In a supplementary analysis, we compared these metrics using just ‘severe’ single-item responses.

#### Longitudinal analysis

To investigate fatigue over time (research question three), we used linear mixed-effects regression to model trajectories in fatigue as assessed using the CFQ. For the primary analyses, we used the total score derived from the bimodal scoring system (and used the Likert-style scoring system in supplementary analysis)^12,13^. We also investigated trajectories in the mental and physical fatigue subscale scores.

The total CFQ score at 3-, 6-, 12-, and 24-months post-infection was our modelled outcome. Our initial model included time since infection, a constant-term, and a participant-level random intercept only. Time was defined as the number of days between the baseline test and questionnaire completion at each follow-up, divided by 30.25 for interpretation in monthly units. We included time as a linear term and explored whether model fit was improved by including other functional forms (square, square root, cube, inverse). Including these forms led to limited improvement according to the Akaike information criterion values compared to the linear model (all differences <9.5; see Supplementary Table 1). Therefore, we retained the more parsimonious linear model and estimated the predicted mean fatigue trajectory with 95% confidence intervals.

To explore if fatigue trajectories varied by participant characteristics, we sequentially added (to the above-described model) explanatory variables, including both fixed main effects and interactions with time. Each variable was tested in a separate model. Explanatory variables were age, sex at birth, ethnicity, IMD quintile, and binary indicators for learning difficulties at school and/or EHCP status, frequent pre-pandemic fatigue, unusual tiredness/fatigue as a main symptom at acute infection, and fulfilment of the PCC definition 3-months post-infection. Likelihood ratio tests were used to compare models with and without the relevant interaction terms to determine if their inclusion significantly improved model fit. For each initial trajectory model (bimodal and Likert-style scoring), we undertook a series of diagnostics which are described in Supplementary Methods and illustrated in Supplementary Figures 1-2.

All analyses were conducted using R (version 4.4.0)^23^ in RStudio. Linear mixed-effects models were constructed using the lmer function from the *lme4 *package^24^. Almost all questions were compulsory in the CLoCk questionnaire and, therefore, within the analytical sample there was no missing data by design. Data from CLoCk are publicly available via the UK Data Service (ID: 9203)^25^. All analyses were pre-specified and exploratory, we therefore did not correct for multiple testing.

## Results

### Sample characteristics

From a total of 31,012 CLoCk participants, 13,690 of whom tested positive for SARS-CoV-2 infection at baseline, we identified 943 participants who tested positive between January-March 2021 and responded to questionnaires at 3-, 6-, 12- and 24-months post-infection. Compared to all baseline test-positive participants, the study cohort was broadly similar demographically and had very similar distributions on baseline fatigue-related variables (Supplementary Table 2). However, the study cohort included more females (68.4% vs. 61.2%) and fewer reporting learning difficulties at baseline (5.6% vs. 7.3%).

Among the study cohort of 943 participants, 581 (61.6%) met CFQ case-ness at least once during follow-up, whilst 362 (38.4%) never reached CFQ case-ness over the follow-up period (Table 1). The ever-cases were more likely to be female (77.1% vs. 54.4%) and older (mean age 15.0 vs. 13.9 years) compared to never-cases. There were limited differences between ever- and never-cases in terms of ethnicity and deprivation, although the proportion residing in the least deprived IMD quintile was lower in ever-cases than never-cases (21.0% vs. 28.2%) (Table 1). At enrolment (in April-June 2021), a higher proportion of ever-cases (vs never-cases) retrospectively reported frequently feeling very tired before the pandemic in early March 2020 (48.7% vs. 18.8%). Ever-cases were more likely to retrospectively report tiredness/fatigue as a main symptom at acute infection (17.4% vs. 9.1%) and to meet the PCC definition 3-months post-infection compared to never-cases (35.6% vs. 7.2%) (Table 1).

**Table 1:** Sample characteristics, stratified by CFQ case-ness* during follow-up: n (%) or mean (SD).

**Characteristic**	**CFQ case ever** N = 581 (61.6%)	**CFQ case never** N = 362 (38.4%)
**Demographics**		
Sex at birth		
Female	448 (77.1%)	197 (54.4%)
Male	133 (22.9%)	165 (45.6%)
Age at infection (years)	15.0 (1.9)	13.9 (2.1)
English Index of Multiple Deprivation		
quintile 1 - most deprived	101 (17.4%)	66 (18.2%)
quintile 2	120 (20.7%)	67 (18.5%)
quintile 3	113 (19.4%)	64 (17.7%)
quintile 4	125 (21.5%)	63 (17.4%)
quintile 5 - least deprived	122 (21.0%)	102 (28.2%)
Ethnicity		
Asian/Asian British	95 (16.4%)	53 (14.6%)
Black/African/Caribbean/British	22 (3.8%)	12 (3.3%)
Mixed	26 (4.5%)	16 (4.4%)
Other	11 (1.9%)	5 (1.4%)
Prefer not to say	2 (0.3%)	3 (0.8%)
White	425 (73.1%)	273 (75.4%)
**Baseline characteristics (reported April-May 2021)**		
EHCP	22 (3.8%)	14 (3.9%)
Learning difficulties at school	38 (6.5%)	15 (4.1%)
Often felt very tired in early March 2020	283 (48.7%)	68 (18.8%)
Unusual fatigue/tiredness reported as main acute infection (in Jan-March 2021) symptom	101 (17.4%)	33 (9.1%)
**PCC prevalence**		
Met research definition 3-months post-infection	207 (35.6%)	26 (7.2%)
Persistently met research definition at all follow-ups	68 (11.7%)	0 (0.0%)

EHCP, Educational Health and Care Plan. PCC: Post-Covid Condition *Case-ness was defined as Chalder Fatigue Scale (CFQ) total score ≥4. Participants were classified as ever meeting CFQ case-ness if they met threshold at any follow-up.

### Profiles of fatigue

**Fig. 1:**
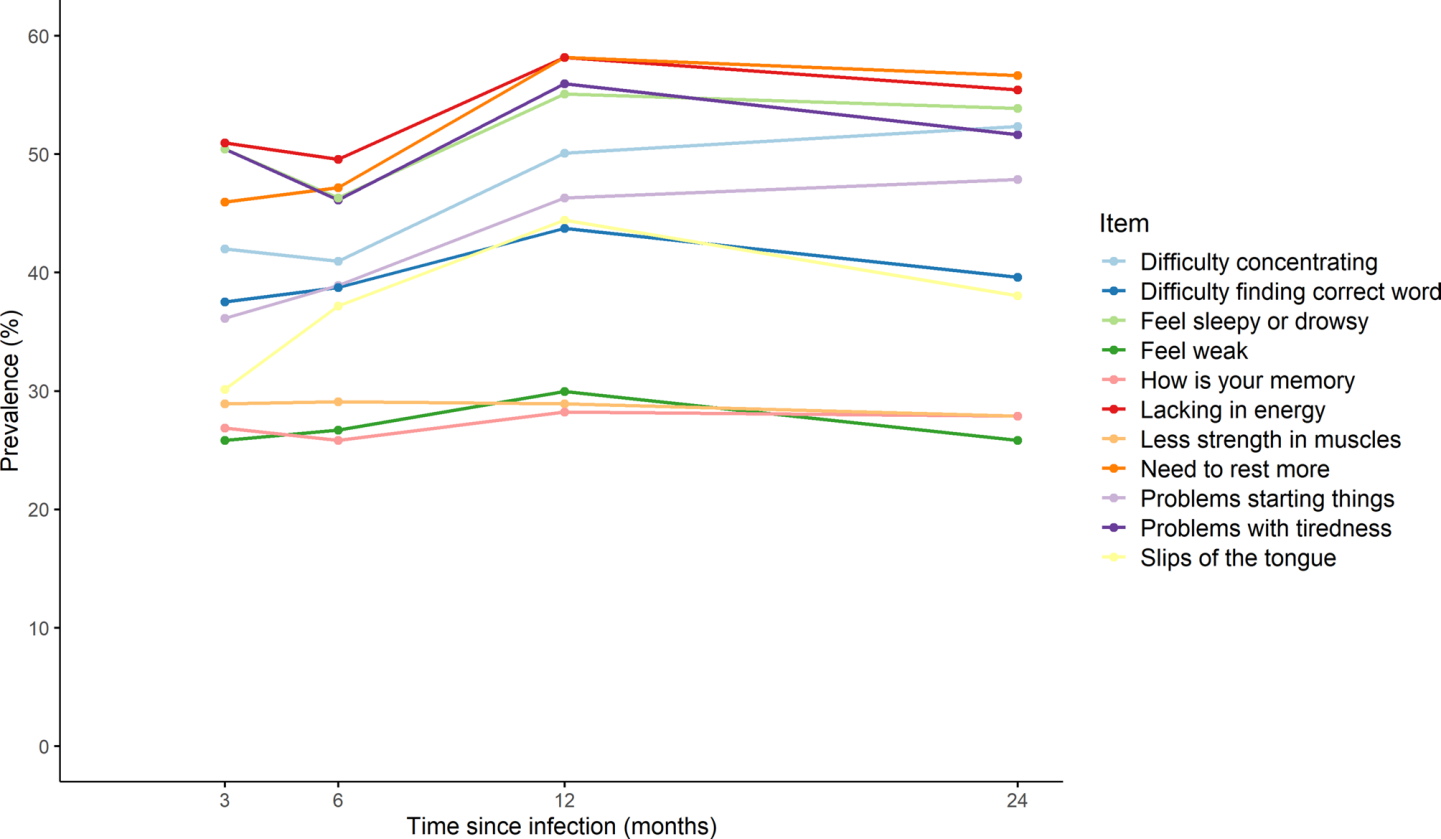
Prevalence of CFQ items over time up to 24 months post-infection among CFQ ever-cases. CFQ, Chalder Fatigue Scale. N=581.

The proportion identified as CFQ cases increased from 35.0% at 3-months to 40.2% at 24-months post-infection (Table 2). Longitudinally, 19.0% met the case-ness threshold just once, 12.6% met the threshold twice, 14.1% three times, and 15.9% persistently at all four follow-ups (Table 2). At all follow ups, mean total scores among never-cases were less than 1 point while they were between 4 and 5 points among ever-cases (Supplementary Table 3). Among ever-cases (n=581), CFQ items relating to lacking in energy, feeling sleepy/drowsy, needing to rest more, and having problems with tiredness had the highest prevalences across follow-ups (Figure 1). CFQ at each time-point demonstrated good reliability with Cronbach’s α ranging from 0.87 at 3- and 6-months to 0.89 at 12-months.

**Table 2:** Proportions of participants meeting CFQ case-ness* threshold cross-sectionally and longitudinally.

	**N (%)**
**Case-ness - cross-sectional**	
At 3m	330 (35.0%)
At 6m	316 (33.5%)
At 12m	391 (41.5%)
At 24m	379 (40.2%)
**Number of times meeting case-ness during follow-up**	
0	362 (38.4%)
1	179 (19.0%)
2	119 (12.6%)
3	133 (14.1%)
Always (i.e. 4 times)	150 (15.9%)
Persistent (i.e. ≥3 times)	283 (30.0%)

N=943. * Case-ness was defined as Chalder Fatigue Scale (CFQ) total score ≥4.

When comparing CFQ case-ness to the single-item fatigue response (mild/severe) across follow-ups, sensitivity ranged between 0.728 and 0.794, specificity between 0.755 and 0.808 and Youden’s J between 0.49 and 0.60. The binarized single-item had positive predictive values ranging from 0.630 to 0.698, and negative predictive values from 0.806 to 0.879 (Table 3). When considering ‘severe fatigue’ alone, sensitivity was lower (≤0.111) but specificity was higher (≥0.989) (Supplementary Table 4). Among participants reporting no unusual fatigue/tiredness on the single-item, between 12.1% and 19.4% were identified as CFQ cases (Supplementary Table 5).

**Table 3:** Performance of mild/severe single-item responses* in fatigue case ascertainment compared to CFQ.

Time-point	N	Number of CFQ Fatigue cases	Total Severe/mild single-item responses	Sensitivity	Specificity	Positive predictive value	Negative predictive value	Youden’s J
3m	943	330	380	0.794	0.808	0.689	0.879	0.601
6m	943	316	387	0.772	0.772	0.630	0.871	0.544
12m	943	391	447	0.798	0.755	0.698	0.841	0.553
24m	943	379	412	0.728	0.759	0.670	0.806	0.487

CFQ, Chalder Fatigue Scale. * ‘Severe’ and ‘mild’ responses to the single-item were combined into a binary variable.

### Fatigue trajectory

**Fig. 2:**
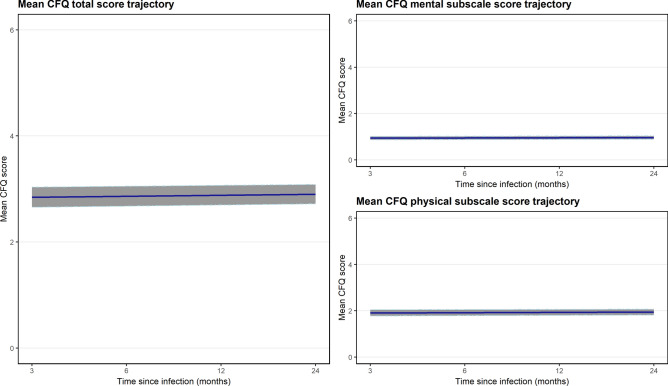
Mean CFQ Trajectory over time: overall and for the mental and physical subscales (95% CI indicated via shading around trajectory).Scored using the Chalder Fatigue Scale (CFQ) bimodal scoring system. N=943.

The CFQ total score increased over time (0.019 points/month, 95% CI, 0.011 to 0.027, *p*<0.001; intra-class correlation: 0.618) (Figure 2). This equated to an increase of 0.448 points (95% CI, 0.252 to 0.645) over 24-months. The mean trajectory was similar when assessed using the Likert-style scoring system (Supplementary Figure 3). For the subscales, on average, physical fatigue scores increased at a rate of 0.012 points/month (95% CI, 0.005 to 0.018, *p*<0.001) while mental fatigue scores increased at a rate of 0.007 points/month (95% CI, 0.004 to 0.010, *p*<0.001) (Figure 2).

**Fig. 3:**
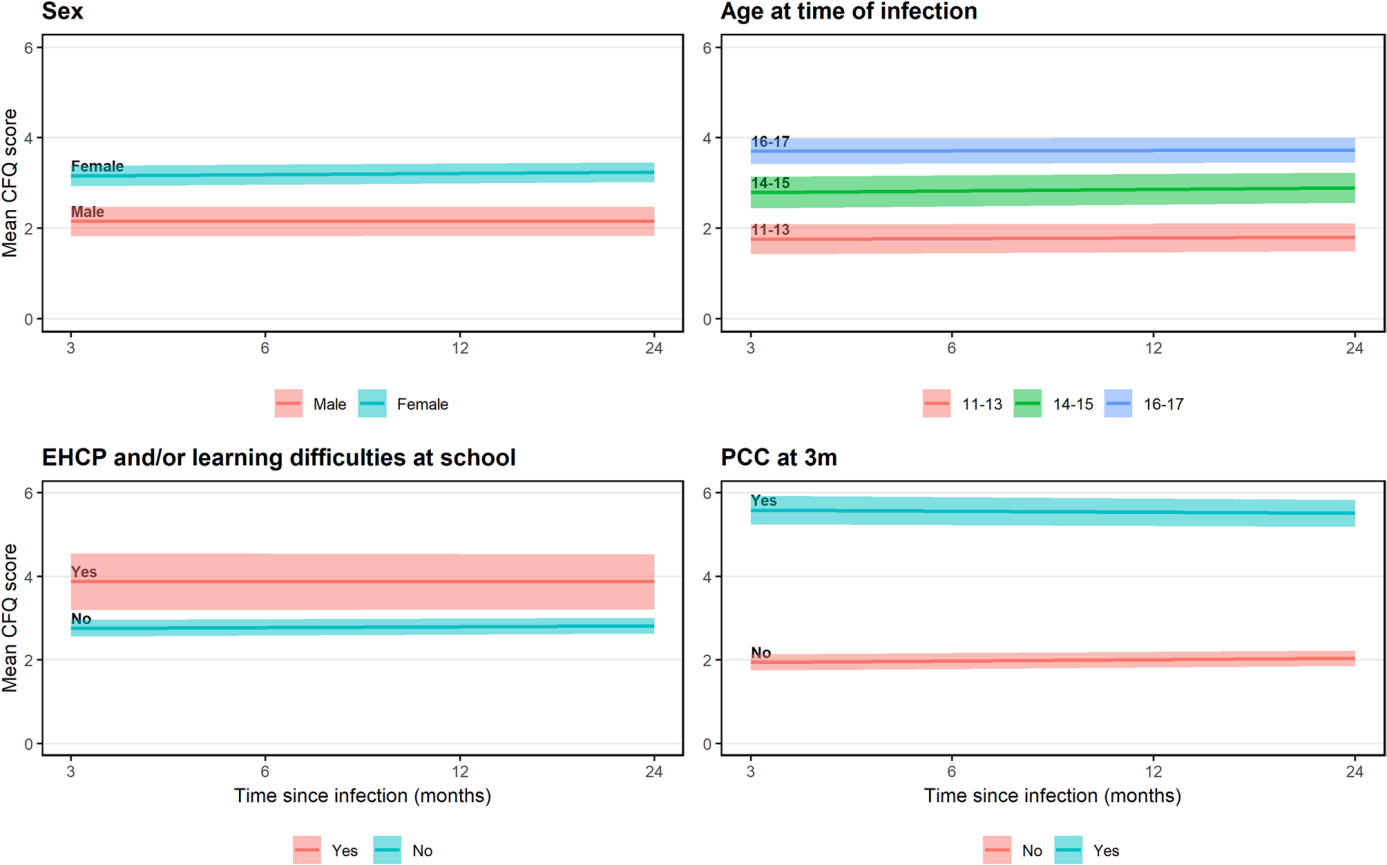
Mean trajectories of CFQ total score, by sex, age at time of infection, EHCP and/or learning difficulties at school, and PCC at 3 m (95% CI indicated via shading around trajectory). CFQ, Chalder Fatigue Scale; PCC, Post-Covid Condition; EHCP, Education Health and Care Plan. N=943.

CFQ total scores differed by sex, age, learning difficulties/EHCP at school, and by whether participants met the PCC definition 3-months post-infection (Figure 3), reported feeling very tired often early in March 2020 (before the pandemic), and reported tiredness/fatigue as a main symptom at acute infection (Supplementary Figure 4). There was less evidence of differences in mean total score by ethnicity and deprivation (Supplementary Figure 4). Mean predicted scores stratified by each characteristic are presented in Supplementary Tables 6-13.

Although small in (absolute) magnitude, the rate of change in total CFQ scores varied by sex (likelihood ratio test interaction *p*=0.004), with a slower rate of change among males compared to females. For example, predicted scores for males increased from 2.16 (95% CI, 1.83 to 2.49) at 3-months to 2.18 (95% CI, 1.82 to 2.53) at 24-months post-infection. Corresponding predicted scores for females increased from 3.16 (95% CI, 2.94 to 3.39) to 3.73 (95% CI, 3.49 to 3.97) (Supplementary Table 6). The rate of change also varied based on whether fatigue/tiredness was reported as a main symptom at testing (interaction *p*<0.01) and whether participants met the PCC definition 3-months post-infection (*p*<0.01). For the former, mean scores tended to converge over time – decreasing among those who reported tiredness/fatigue as a main symptom and increasing among those who did not (Supplementary Table 12). For the latter, scores decreased over time for participants that met the PCC definition, while they increased for those who did not. By 24-months, those that met the PCC definition had a predicted mean score almost double that of those who did not (5.04 vs. 2.65) (Supplementary Table 13). Model diagnostics indicated that, for the bimodal scoring model (Supplementary Figure 1), residuals were close to normal and random intercepts only slightly skewed. In contrast, residuals and random intercepts from the Likert scoring system (Supplementary Figure 2) showed clear departures from normality.

## Discussion

In this longitudinal analysis involving 943 CYP in England followed up to 24 months after SARS-CoV-2 infection, we found that, at each time-point, over a third were identified as cases on the CFQ, and almost one in six persistently met case-ness over the entire follow-up period. Ever-cases were more likely to be female, older, report pre-pandemic fatigue, and meet the PCC definition 3-months post-infection than never-cases.

We observed a small-to-moderate overall increase in fatigue over time in the 943 participants, with CFQ total scores increasing by 0.448 points on average (95% CI, 0.252 to 0.645) over 24-months. However, scores differed by specific characteristics. Females had higher scores than males, older CYP had higher scores than younger CYP, and those who reported learning difficulties at school had higher scores than those without. Participants that met PCC definition 3-months post-testing had higher scores than those who did not, and those who reported fatigue prior to the pandemic and as their main symptom at acute infection remained more fatigued during follow-up than their counterparts. We found limited evidence to suggest that fatigue varied by ethnicity or deprivation, although small samples in some of the individual categories may have reduced the power of our analysis. Additionally, the rate of change in CFQ scores over time was faster in females (with fatigue worsening over time), compared to males (where scores were relatively constant). These sex-differences might be attributable to differences in hormones, comorbidities, and pain sensitivity^26^. These hypotheses therefore require further investigation to inform targeted interventions. The rate of change was also slower in those reporting fatigue/tiredness as a main symptom at testing or meeting PCC definition at 3-months post-infection – improving slightly over time – compared to those who did not, in whom scores worsened. These results suggest that fatigue remains relatively persistent once established, while those without early fatigue are at risk of deterioration, underscoring a need for greater preventative efforts. Given that the bimodal total fatigue score scale ranges from 0-to-11, it is important to note that observed differences over time (e.g., by 0.448 points on average over 24-months) were modest and unlikely to impact the identification of case-ness. Future work is needed to determine the clinical significance of these differences – namely, to estimate minimal clinically important differences (similar to those established for adults^27^) – and to explore transition probabilities (e.g., remission vs. incident case-ness). Further, while we considered standard functional forms of time in our modelling, future work might explore more flexible approaches (e.g., splines). Notwithstanding, our results may be useful in identifying CYP most likely to experience post-infection fatigue. In turn, this can help inform more targeted intervention efforts.

A unique aspect of the CLoCk study was the assessment of fatigue using both a reliable and valid scale as well as a single-item assessment^12,13^. The latter is appealing for assessing many symptoms at once, but there is a risk that such items may not be sufficient for case ascertainment. Participants were asked, “Are you experiencing unusual fatigue/tiredness?” with response options corresponding to no, mild, and severe fatigue. Combining mild and severe responses^1,8^ and comparing them to CFQ case-ness as a benchmark, sensitivity and specificity were ≥0.728 and negative predictive values (≥0.806) were higher than positive predictive values (≥0.630) over follow-up. This suggests that single-item assessment is a useful, practical tool to briefly assess mild/severe fatigue in CYP post-infection. However, cross-tabulation of the single-item and CFQ case-ness highlighted that, across follow-ups, between 12% and 19% of those reporting no fatigue/tiredness on the single-item were identified as CFQ cases. While we did not expect near-perfect convergence, this finding suggests that the single-item has a reduced ability to detect borderline cases, which may be of particular importance when assessing symptom emergence or subclinical manifestations. The single-item might be improved by using a greater number of response options and/or providing additional examples of how fatigue might manifest to improve participant understanding. Ultimately, the choice of measures for future studies and in clinical services will depend on whether the main objective is to detect fatigue with minimal burden or to comprehensively assess the full spectrum of fatigue severity, with the latter requiring more detailed measures such as the CFQ. Relatedly, our study drew participants from the general population and so results might not generalise to patients seen in clinical services. Thus, additional validation work would be needed to inform clinical adoption.

Another important aspect to consider when interpreting evidence on symptoms following SARS-CoV-2 infection is the extent to which such symptoms were present in CYP pre-pandemic and/or pre-infection. Specifically, it is necessary to distinguish the extent to which post-infection fatigue differs from fatigue generally reported by CYP^28^. A systematic synthesis of international pre-pandemic data sources on adolescents identified that several symptoms commonly reported post-infection, including headache, cough, fatigue, and pain, had high prevalence in CYP pre-pandemic: specifically, 21.5% for fatigue^28^. In the UK, a 1999 investigation^5^ into fatigue assessed 842 adolescents (11-to-15-years) at baseline and 4–6 months later and classified participants as fatigued if answering affirmatively to the question, “Over the last month, have you been feeling much more tired and worn out than usual?” Authors reported a fatigue prevalence of 34.1% (95% CI, 30.9 to 37.3) at baseline and 38.1% (95% CI, 34.8 to 41.5) at follow-up. These figures are remarkably similar to the 35% we detected as CFQ cases 3-months after infection, which increased to 40% by 24-months. Taken together, these findings suggests that the proportion of fatigued participants after infection is not substantially different to what might have been expected despite the pandemic, notwithstanding differences in ascertainment methods, study time periods, and exposure to SARS-CoV-2 in our sample. This raises important questions about whether fatigue can have diagnostic specificity for PCC, given the potential for pre-existing fatigue to be misattributed as post-SARS-CoV-2 sequelae. Despite similar prevalence, however, these results do not tell us whether fatigue after infection qualitatively differs to pre-existing or pre-pandemic fatigue – such investigations are warranted to inform efforts to improve the detection and diagnosis of PCC among CYP.

Although distinct, the overlap between paediatric chronic fatigue syndrome and PCC in terms of fatigue and other symptoms is striking, with some authors tentatively suggesting that SARS-CoV-2 infection could trigger post-infectious fatigue syndrome: not dissimilar to outcomes following other serious viruses (including earlier coronaviruses and meningitis)^29^. We reported mean CFQ scores ranging from 4.25 to 5.00 among CFQ cases across follow-ups – comparable to 4.38 reported from a cross-sectional sample of 36 CYP attending a specialist chronic fatigue syndrome clinic in South East England^30^. Detailed studies characterising the phenotypic features of these two diagnoses will aid understanding of their similarities and differences. Further understanding of fatigue in PCC in CYP may also be obtained by consideration of fatigue in PCC in adults. Meta-analyses indicate that one third of adults experience persistent fatigue following SARS-CoV-2 infection^31^, and that fatigue is associated with some non-modifiable factors, including female sex and age, and modifiable factors, such as mental health, specifically anxiety, depression and post- traumatic stress^32^. This study examined experiences of fatigue trajectories to enhance understanding of the natural progression of fatigue over time since SARS-CoV-2 infection, and future research is needed to explore direct and indirect (potentially bi-directional) pathways linking fatigue and mental health among CYP^33^over time. The potential pathophysiological mechanisms for post-viral fatigue are currently unknown for both adults and CYP, but are likely to be multifactorial, resulting from the dysregulation of multiple systems in response to a particular trigger^34^.

This study has a number of strengths. We included a large sample of almost 1,000 CYP with definitive ascertainment of SARS-CoV-2 positive status at baseline, with follow-up to 24-months post-infection. We assessed fatigue using a valid and reliable scale, enabling robust characterisation of fatigue experiences and improving on the single-item assessment of fatigue/tiredness used in previous studies based on the CLoCk sample^1,8^as well as others^9,10^. Our primary analyses used the CFQ bimodal scoring system and our findings were robust as we saw similar overall results using the Likert-style scoring system.

This study also has several limitations. First, although we had definitive ascertainment of SARS-CoV-2 positive status at study invitation, we did not have reliable data on reinfections, as mass national testing ceased in early 2023. Therefore we cannot comment on whether some participants’ fatigue was related to later infections. This is also why we did not include a comparison group of participants testing negative for SARS-CoV-2 at baseline. Including a comparison group would have improved the methodological rigour of our study^11^, but progression of the pandemic made this practically impossible given many such participants may have been infected or developed antibodies at some point during follow-up^35^. Second, we do not know if participants’ fatigue was related to any other health issues or life events^11^nor what participants’ experiences of fatigue were between follow-ups and if it was modified by vaccination. Although we have conducted four follow-ups to date, it is possible that the schedule was too infrequent to identify fluctuating symptom trajectories. Studies employing ecological momentary assessment could be valuable here; however, it is important to balance the burden of questionnaire completion for participants, and additional follow-ups might have led to greater attrition. Third, we included participants who responded to questionnaires at all follow-up time-points. This potentially induced some attrition and/or selection bias as we observed that, compared to all CLoCk participants who tested positive for SARS-CoV-2 at baseline, our study cohort contained more females and slightly fewer participants reporting learning difficulties. This sample has also previously been shown to include more females and least deprived CYP compared to the target population of CYP testing positive for SARS-CoV-2 between September 2020 to March 2021 in England^15^. Reasons for the potential biases include, for example, CYP with symptoms to report being more engaged with the study and those with learning difficulties potentially needing additional support to complete the measures. However, it was reassuring that the distributions for baseline fatigue-related variables were almost identical between our study cohort and all baseline-positive CLoCk participants. Understanding the representativeness of the CLoCk sample, developing and testing flexible weighting strategies, is an area of important and ongoing research^36^. Fourth, we studied fatigue trajectories stratified by whether participants met the research definition for PCC. Given the definition used, we acknowledge that some participants meeting the PCC definition could be experiencing fatigue which was impacting their daily lives. Fifth, we described CFQ single-item responses, but it should be noted that the scale was not designed for single-item analysis and was included solely to illustrate participants’ experiences of fatigue. Finally, some subgroup-defining variables, such as whether participants often felt very tired before the pandemic and their main symptoms at acute infection, were retrospectively reported 3-months post-infection and are subject to recall bias. Additionally, for pre-pandemic characteristics, we were unable to specify when/how long before the pandemic these characteristics presented.

## Conclusion

Persistent fatigue is prominent in CYP up to 24-months after SARS-CoV-2 infection. Subgroup differences in scores and trajectories highlight the need for targeted interventions. Single-item assessment of fatigue is a useful practical tool to detect potential severe fatigue.

## Supplementary Information

Supplementary Information.

## Data Availability

Data from the Children and Young People with Long COVID (CLoCk) study are publicly available via the UK Data Service (ID: 9203).
