# Supplementary material for: Fatigue in children and young people up to 24 months after infection with SARS-CoV-2
Source: Sci Rep. 2025 Nov 20;15:41105. doi: 10.1038/s41598-025-24868-x (PMC12635370; doi:10.1038/s41598-025-24868-x)
Supplement: Supplementary file 1 — Supplementary Information. [file 41598_2025_24868_MOESM1_ESM.docx]

Supplement to

**“Fatigue in children and young people up to 24 months**
**after infection with SARS-CoV-2”**

Alvin Richards-Belle, Roz Shafran, Natalia K. Rojas, Terence Stephenson, Ewan Carr, Trudie Chalder, Emma Dalrymple, Kelsey McOwat, Ruth Simmons, CLoCk Consortium, Snehal M. Pinto Pereira

[Supplementary Methods 2](#_Toc209959247)

[Supplementary Tables 3](#_Toc209959248)

[Supplementary Table 1. Comparison of functional forms of time using AIC. 3](#_Toc209959249)

[Supplementary Table 2. Baseline characteristics of all CLoCk participants, those testing positive for SARS-CoV-2 at baseline, and the sub-cohort included in the present study. 4](#_Toc209959250)

[Supplementary Table 3. Mean (SD) CFQ total and subscale scores. 5](#_Toc209959251)

[Supplementary Table 4. Performance of severe single-item responses* in fatigue case ascertainment compared to CFQ. 6](#_Toc209959252)

[Supplementary Table 5. Cross-sectional cross-tabulation of single-item assessments and CFQ case-ness* at each follow-up. 7](#_Toc209959253)

[Supplementary Table 6. Predicted CFQ scores over time, stratified by sex. 8](#_Toc209959254)

[Supplementary Table 7. Predicted CFQ scores over time, stratified by age group at baseline testing. 9](#_Toc209959255)

[Supplementary Table 8. Predicted CFQ scores over time, stratified by ethnicity. 10](#_Toc209959256)

[Supplementary Table 9. Predicted CFQ scores over time, stratified by quintile of the English Index of Multiple Deprivation. 11](#_Toc209959257)

[Supplementary Table 10. Predicted CFQ scores over time, stratified by Education Health and Care Plan and/or learning difficulties at school (reported at baseline). 12](#_Toc209959258)

[Supplementary Table 11. Predicted CFQ scores over time, stratified by feeling tired very often early in March 2020 (reported at baseline). 13](#_Toc209959259)

[Supplementary Table 12. Predicted CFQ scores over time, stratified by tiredness/fatigue as main symptom at acute infection (reported at baseline). 14](#_Toc209959260)

[Supplementary Table 13. Predicted CFQ scores over time, stratified by meeting Post-COVID condition definition at three months post-infection. 15](#_Toc209959261)

[Supplementary Figures 16](#_Toc209959262)

[Supplementary Figure 1. CFQ trajectory (bimodal scoring system) model diagnostics. 16](#_Toc209959263)

[Supplementary Figure 2. CFQ trajectory (Likert-style scoring system) model diagnostics. 17](#_Toc209959264)

[Supplementary Figure 3. Trajectory of CFQ total score over time – scored according to the Likert-style scale scoring system (95% CI indicated via shading around trajectory). 18](#_Toc209959265)

[Supplementary Figure 4. Trajectory of CFQ total score, by ethnicity, IMD quintile, feeling of tiredness very often in early in March 2020, and tiredness/fatigue as main acute infection symptom (95% CI indicated via shading around trajectory). 19](#_Toc209959266)

# Supplementary Methods

For each initial trajectory model (bimodal and Likert-style scoring), we examined underlying assumptions. Specifically, we: (i) visually assessed normality of residuals using a Quantile-Quantile plot, (ii) visually assessed homoscedasticity of residuals by plotting them against fitted values (i.e., fixed and predicted random effects) and (iii) visually assessed normality of random intercepts using Quantile-Quantile plots. For each model, plots can be seen in Supplementary Figures 1-2.

# Supplementary Tables

## Supplementary Table 1. Comparison of functional forms of time using AIC.

|  | **AIC** | **Difference** |
| --- | --- | --- |
| Linear (reference) | 17899.71 | - |
| Linear term and additional term: | | |
| Square (time^2^) | 17902.86 | +3.15 |
| Square root (√time) | 17894.91 | –4.81 |
| Cubic (time^3^) | 17909.12 | +9.41 |
| Inverse (1/time) | 17896.94 | –2.77 |

*AIC, akaike information criterion.*

## Supplementary Table 2. Baseline characteristics of all CLoCk participants, those testing positive for SARS-CoV-2 at baseline, and the sub-cohort included in the present study.

| Characteristic | All CLoCk participants  N = 31,012 | Baseline test-positive CLoCk participants N = 13,690 | Study cohort*  N = 943 |
| --- | --- | --- | --- |
| **Demographics** |  |  |  |
| Sex at birth |  |  |  |
| Female | 19,051 (61.4%) | 8,385 (61.2%) | 645 (68.4%) |
| Male | 11,961 (38.6%) | 5,305 (38.8%) | 298 (31.6%) |
| Age at infection (years) | 14.4 (1.9) | 14.4 (1.9) | 14.6 (2.0) |
| English Index of Multiple Deprivation |  |  |  |
| quintile 1 - most deprived | 5,345 (17.2%) | 2,416 (17.6%) | 167 (17.7%) |
| quintile 2 | 5,548 (17.9%) | 2,477 (18.1%) | 187 (19.8%) |
| quintile 3 | 5,792 (18.7%) | 2,509 (18.3%) | 177 (18.8%) |
| quintile 4 | 6,656 (21.5%) | 2,917 (21.3%) | 188 (19.9%) |
| quintile 5 - least deprived | 7,671 (24.7%) | 3,371 (24.6%) | 224 (23.8%) |
| Ethnicity |  |  |  |
| Asian | 4,553 (14.7%) | 2,112 (15.4%) | 148 (15.7%) |
| Black | 933 (3.0%) | 390 (2.8%) | 34 (3.6%) |
| Mixed | 1,615 (5.2%) | 681 (5.0%) | 42 (4.5%) |
| Other | 524 (1.7%) | 264 (1.9%) | 16 (1.7%) |
| Prefer not to say | 189 (0.6%) | 88 (0.6%) | 5 (0.5%) |
| White | 23,198 (74.8%) | 10,155 (74.2%) | 698 (74.0%) |
| Region |  |  |  |
| East Midlands | 2,210 (7.1%) | 1,051 (7.7%) | 92 (9.8%) |
| East of England | 6,047 (19.5%) | 2,211 (16.2%) | 139 (14.7%) |
| London | 6,157 (19.9%) | 2,618 (19.1%) | 137 (14.5%) |
| North East | 1,198 (3.9%) | 596 (4.4%) | 31 (3.3%) |
| North West | 3,606 (11.6%) | 1,711 (12.5%) | 111 (11.8%) |
| South East | 4,917 (15.9%) | 2,167 (15.8%) | 169 (17.9%) |
| South West | 1,514 (4.9%) | 742 (5.4%) | 88 (9.3%) |
| West Midlands | 3,032 (9.8%) | 1,431 (10.5%) | 118 (12.5%) |
| Yorkshire and The Humber | 2,331 (7.5%) | 1,163 (8.5%) | 58 (6.2%) |
| **Baseline characteristics (reported April-May 2021)** |  |  |  |
| Education Health and Care Plan | 1,625 (5.2%) | 617 (4.5%) | 36 (3.8%) |
| Learning difficulties at school | 2,478 (8.0%) | 1,005 (7.3%) | 53 (5.6%) |
| Often felt tired in early March 2020 | 12,001 (38.7%) | 5,064 (37.0%) | 351 (37.2%) |
| Unusual fatigue/tiredness reported as main acute infection (in Jan-March 2021) symptom | 2,387 (7.7%) | 1,655 (12.1%) | 134 (14.2%) |
| **Fatigue at 3m after infection (CFQ)** |  |  |  |
| Case-ness^1^ | 10,416 (33.6%) | 4,847 (35.4%) | 330 (35.0%) |
| Total score | 2.7 (3.1) | 2.9 (3.1) | 2.8 (3.1) |
| Physical subscale score | 1.8 (2.2) | 1.9 (2.3) | 1.9 (2.2) |
| Mental subscale score | 0.9 (1.2) | 0.9 (1.2) | 0.9 (1.2) |

* baseline test positive CLoCk participants with data at 3-, 6,-,12- and 24-month after infection.

*^1^ Case-ness was defined as Chalder Fatigue Scale (CFQ) total score ≥4. Numbers and proportions are presented for categorical variables, means with standard deviations are presented for continuous variables.*

## Supplementary Table 3. Mean (SD) CFQ total and subscale scores.

| **Characteristic** | **CFQ case ever**  N = 581 | **CFQ case never**  N = 362 |
| --- | --- | --- |
| *3m* |  |  |
| Total score | 4.25 (3.18) | 0.50 (0.87) |
| Physical fatigue subscale score | 2.89 (2.32) | 0.31 (0.66) |
| Mental fatigue subscale score | 1.36 (1.34) | 0.19 (0.51) |
| *6m* |  |  |
| Total score | 4.27 (3.18) | 0.46 (0.88) |
| Physical fatigue subscale score | 2.84 (2.33) | 0.30 (0.68) |
| Mental fatigue subscale score | 1.43 (1.38) | 0.15 (0.47) |
| *12m* |  |  |
| Total score | 5.00 (3.2) | 0.50 (0.9) |
| Physical fatigue subscale score | 3.33 (2.37) | 0.32 (0.67) |
| Mental fatigue subscale score | 1.66 (1.39) | 0.17 (0.46) |
| *24m* |  |  |
| Total score | 4.80 (3.2) | 0.50 (0.9) |
| Physical fatigue subscale score | 3.19 (2.38) | 0.35 (0.71) |
| Mental fatigue subscale score | 1.58 (1.42) | 0.20 (0.54) |

*CFQ, Chalder Fatigue Scale.*

## Supplementary Table 4. Performance of severe single-item responses* in fatigue case ascertainment compared to CFQ.

| Time-point | N | Number of CFQ Fatigue cases | Total Severe single-item responses | Sensitivity | Specificity | Positive predictive value | Negative predictive value | Youden’s J |
| --- | --- | --- | --- | --- | --- | --- | --- | --- |
| 3m | 943 | 330 | 35 | 0.094 | 0.993 | 0.886 | 0.671 | 0.087 |
| 6m | 943 | 316 | 34 | 0.098 | 0.995 | 0.912 | 0.686 | 0.093 |
| 12m | 943 | 391 | 49 | 0.110 | 0.989 | 0.878 | 0.611 | 0.099 |
| 24m | 943 | 379 | 45 | 0.111 | 0.995 | 0.933 | 0.625 | 0.105 |

*CFQ, Chalder Fatigue Scale.* ‘Severe’ responses to the single-item were treated as a binary variable.*

## Supplementary Table 5. Cross-sectional cross-tabulation of single-item assessments and CFQ case-ness* at each follow-up.

|  |  | **Single-item assessment** |  |
| --- | --- | --- | --- |
|  | **No fatigue** | **Mild fatigue** | **Severe fatigue** |
| **At 3m** | n=563 | n=345 | n=35 |
| CFQ case | 68 (12.1%) | 231 (67.0%) | 31 (88.6%) |
| Non-case | 495 (87.9%) | 114 (33.0%) | 4 (11.4%) |
| **At 6m** | n=556 | n=353 | n=34 |
| CFQ case | 72 (12.9%) | 213 (60.3%) | 31 (91.2%) |
| Non-case | 484 (87.1%) | 140 (39.7%) | 3 (8.8%) |
| **At 12m** | n=496 | n=398 | n=49 |
| CFQ case | 79 (15.9%) | 269 (67.6%) | 43 (87.8%) |
| Non-case | 417 (84.1%) | 129 (32.4%) | 6 (12.2%) |
| **At 24m** | n=531 | n=367 | n=45 |
| CFQ case | 103 (19.4%) | 234 (63.8%) | 42 (93.3%) |
| Non-case | 428 (80.6%) | 133 (36.2%) | 3 (6.7%) |

*N=943. Case-ness was defined as Chalder Fatigue Scale (CFQ) total score ≥4.*

## Supplementary Table 6. Predicted CFQ scores over time, stratified by sex.

| **CFQ total score,  time-point** | **Female** N = 645 | **Male** N = 298 |
| --- | --- | --- |
| 3m | 3.16 (2.94, 3.39) | 2.16 (1.83, 2.49) |
| 6m | 3.24 (3.03, 3.46) | 2.16 (1.84, 2.48) |
| 12m | 3.40 (3.20, 3.61) | 2.16 (1.86, 2.47) |
| 24m | 3.73 (3.49, 3.97) | 2.18 (1.82, 2.53) |

*Predicted CFQ total scores (95% confidence interval) derived from a linear mixed-effects model with CFQ total score as the outcome. Predictors included time, sex, and their interaction. A random intercept for participant was included. A likelihood ratio test comparing models with and without the interaction term yielded p=0.004.*

## Supplementary Table 7. Predicted CFQ scores over time, stratified by age group at baseline testing.

| **CFQ total score,  time-point** | **11-13** N = 293 | **14-15** N = 261 | **16-17** N = 389 |
| --- | --- | --- | --- |
| 3m | 1.76 (1.43, 2.09) | 2.79 (2.44, 3.14) | 3.70 (3.42, 3.99) |
| 6m | 1.81 (1.49, 2.12) | 2.90 (2.57, 3.23) | 3.73 (3.46, 4.00) |
| 12m | 1.90 (1.60, 2.20) | 3.12 (2.80, 3.44) | 3.78 (3.52, 4.04) |
| 24m | 2.09 (1.74, 2.44) | 3.56 (3.19, 3.93) | 3.88 (3.58, 4.19) |

*Predicted CFQ total scores (95% confidence interval) derived from a linear mixed-effects model with CFQ total score as the outcome. Predictors included time, age (continuous), and their interaction. A random intercept for participant was included. A likelihood ratio test comparing models with and without the interaction term yielded p=0.308.*

## Supplementary Table 8. Predicted CFQ scores over time, stratified by ethnicity.

| **CFQ total score,  time-point** | **Asian/Asian British** N = 148 | **Black/African/ Caribbean/ British** N = 34 | **Mixed/Other** N = 58 | **White** N = 698 |
| --- | --- | --- | --- | --- |
| 3m | 2.78 (2.30, 3.26) | 2.79 (1.78, 3.79) | 3.05 (2.29, 3.82) | 2.85 (2.63, 3.07) |
| 6m | 2.89 (2.43, 3.35) | 2.80 (1.84, 3.76) | 3.09 (2.36, 3.82) | 2.90 (2.69, 3.11) |
| 12m | 3.11 (2.67, 3.56) | 2.82 (1.89, 3.74) | 3.17 (2.46, 3.88) | 3.00 (2.79, 3.20) |
| 24m | 3.55 (3.04, 4.06) | 2.86 (1.79, 3.93) | 3.33 (2.51, 4.15) | 3.19 (2.96, 3.43) |

*Predicted CFQ total scores (95% confidence interval) derived from a linear mixed-effects model with CFQ total score as the outcome. Predictors included time, ethnicity, and their interaction. A random intercept for participant was included. A likelihood ratio test comparing models with and without the interaction term yielded p=0.296. Interaction term P values for individual ethnicity levels and time were as follows: Asian/Asian British (0.082), Black/African/Caribbean/British (0.565), and Mixed/Other (0.847). Note that ‘White’ was taken as the reference level.*

## Supplementary Table 9. Predicted CFQ scores over time, stratified by quintile of the English Index of Multiple Deprivation.

| **CFQ total score,  time-point** | **Quintile 1 -  most deprived** N = 167 | **Quintile 2** N = 187 | **Quintile 3** N = 177 | **Quintile 4** N = 188 | **Quintile 5 -  least deprived** N = 224 |
| --- | --- | --- | --- | --- | --- |
| 3m | 3.16 (2.71, 3.60) | 2.63 (2.21, 3.05) | 3.28 (2.84, 3.71) | 2.94 (2.52, 3.37) | 2.37 (1.98, 2.75) |
| 6m | 3.15 (2.72, 3.58) | 2.72 (2.31, 3.12) | 3.32 (2.90, 3.74) | 2.99 (2.59, 3.40) | 2.46 (2.09, 2.83) |
| 12m | 3.14 (2.72, 3.55) | 2.89 (2.50, 3.28) | 3.40 (3.00, 3.81) | 3.10 (2.70, 3.49) | 2.65 (2.29, 3.00) |
| 24m | 3.11 (2.63, 3.59) | 3.23 (2.78, 3.69) | 3.58 (3.11, 4.04) | 3.30 (2.84, 3.75) | 3.02 (2.60, 3.43) |

*Predicted CFQ total scores (95% confidence interval) derived from a linear mixed-effects model with CFQ total score as the outcome. Predictors included time, Index of Multiple Deprivation, and their interaction. A random intercept for participant was included. A likelihood ratio test comparing models with and without the interaction term yielded p=0.094. Interaction term P values for individual deprivation levels and time were as follows: Quintile 1 – most deprived (0.011), Quintile 2 (0.863), Quintile 3 (0.197), and Quintile 4 (0.261). Note that ‘Quintile 5 – least deprived’ was taken as the reference level.*

## Supplementary Table 10. Predicted CFQ scores over time, stratified by Education Health and Care Plan and/or learning difficulties at school (reported at baseline).

| **CFQ total score,  time-point** | **No** N = 871 | **Yes** N = 72 |
| --- | --- | --- |
| 3m | 2.76 (2.56, 2.96) | 3.88 (3.20, 4.56) |
| 6m | 2.82 (2.63, 3.01) | 3.87 (3.22, 4.53) |
| 12m | 2.94 (2.76, 3.13) | 3.86 (3.23, 4.49) |
| 24m | 3.19 (2.98, 3.40) | 3.84 (3.10, 4.57) |

*Predicted CFQ total scores (95% confidence interval) derived from a linear mixed-effects model with CFQ total score as the outcome. Predictors included time, EHCP/learning difficulties, and their interaction. A random intercept for participant was included. A likelihood ratio test comparing models with and without the interaction term yielded p=0.150.*

## Supplementary Table 11. Predicted CFQ scores over time, stratified by feeling tired very often early in March 2020 (reported at baseline).

| **CFQ total score,  time-point** | **No** N = 592 | **Yes** N = 351 |
| --- | --- | --- |
| 3m | 1.96 (1.74, 2.18) | 4.34 (4.05, 4.63) |
| 6m | 2.02 (1.81, 2.23) | 4.39 (4.11, 4.66) |
| 12m | 2.14 (1.94, 2.34) | 4.48 (4.22, 4.74) |
| 24m | 2.39 (2.15, 2.63) | 4.67 (4.36, 4.98) |

*Predicted CFQ total scores (95% confidence interval) derived from a linear mixed-effects model with CFQ total score as the outcome. Predictors included time, feeling tired very often early in March 2020, and their interaction. A random intercept for participant was included. A likelihood ratio test comparing models with and without the interaction term yielded p=0.577.*

## Supplementary Table 12. Predicted CFQ scores over time, stratified by tiredness/fatigue as main symptom at acute infection (reported at baseline).

| **CFQ total score,  time-point** | **No** N = 809 | **Yes** N = 134 |
| --- | --- | --- |
| 3m | 2.58 (2.38, 2.78) | 4.46 (3.96, 4.95) |
| 6m | 2.66 (2.47, 2.86) | 4.34 (3.86, 4.82) |
| 12m | 2.83 (2.64, 3.02) | 4.11 (3.65, 4.57) |
| 24m | 3.17 (2.95, 3.39) | 3.64 (3.11, 4.17) |

*Predicted CFQ total scores (95% confidence interval) derived from a linear mixed-effects model with CFQ total score as the outcome. Predictors included time, tiredness/fatigue as main symptom at acute infection, and their interaction. A random intercept for participant was included. A likelihood ratio test comparing models with and without the interaction term yielded p<0.001.*

## Supplementary Table 13. Predicted CFQ scores over time, stratified by meeting Post-COVID condition definition at three months post-infection.

| **CFQ total score,  time-point** | **No** N = 710 | **Yes** N = 233 |
| --- | --- | --- |
| 3m | 1.94 (1.75, 2.14) | 5.59 (5.25, 5.93) |
| 6m | 2.04 (1.86, 2.23) | 5.51 (5.19, 5.83) |
| 12m | 2.24 (2.07, 2.42) | 5.35 (5.05, 5.66) |
| 24m | 2.65 (2.44, 2.86) | 5.04 (4.67, 5.41) |

*Predicted CFQ total scores (95% confidence interval) derived from a linear mixed-effects model with CFQ total score as the outcome. Predictors included time, Post-COVID condition definition at three months, and their interaction. A random intercept for participant was included. A likelihood ratio test comparing models with and without the interaction term yielded p<0.001.*

# Supplementary Figures


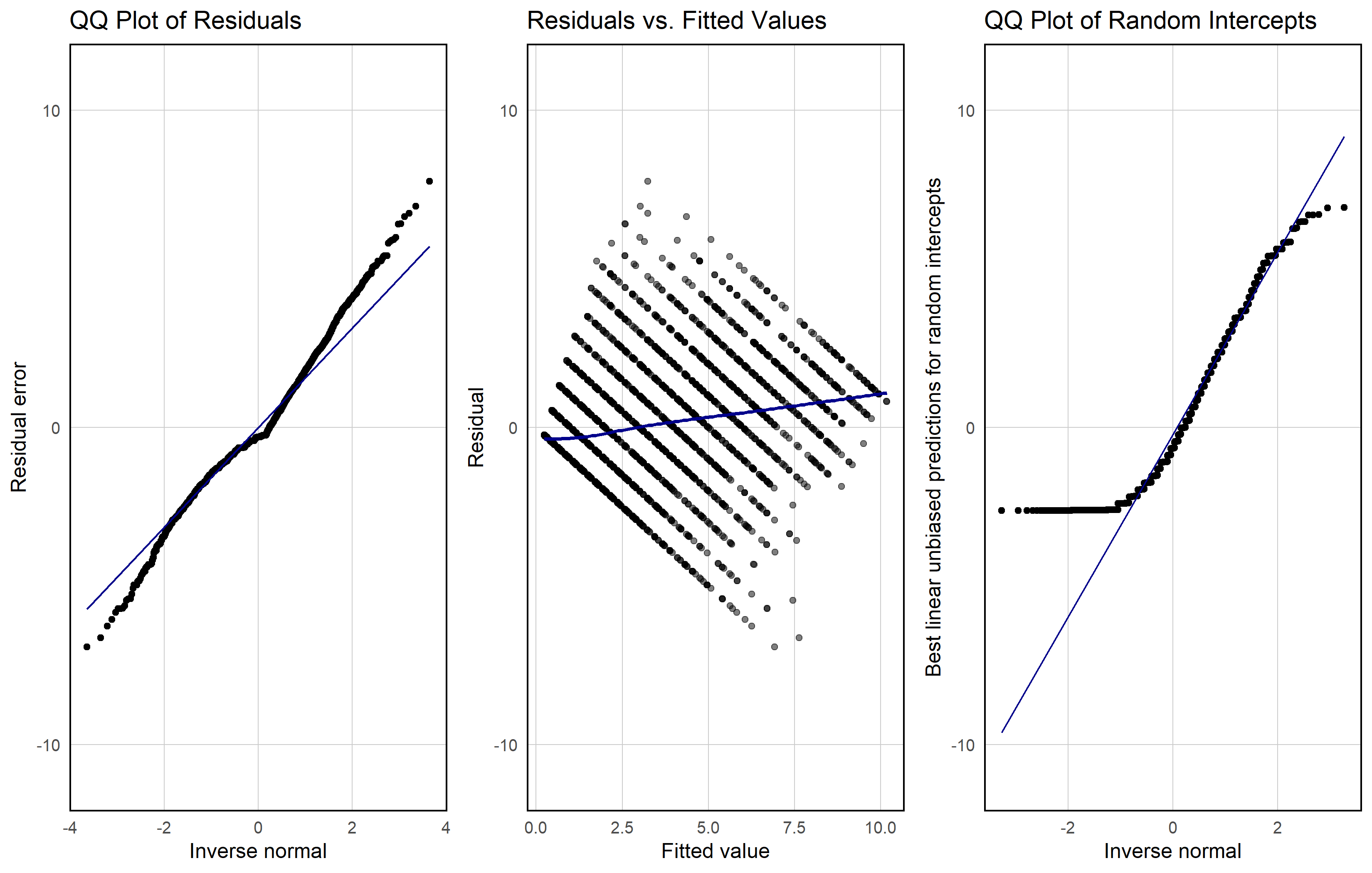


## Supplementary Figure 1. CFQ trajectory (bimodal scoring system) model diagnostics.

*The initial model included CFQ total score (bimodal scoring system) with a linear term for time since infection and a participant-level random intercept.*


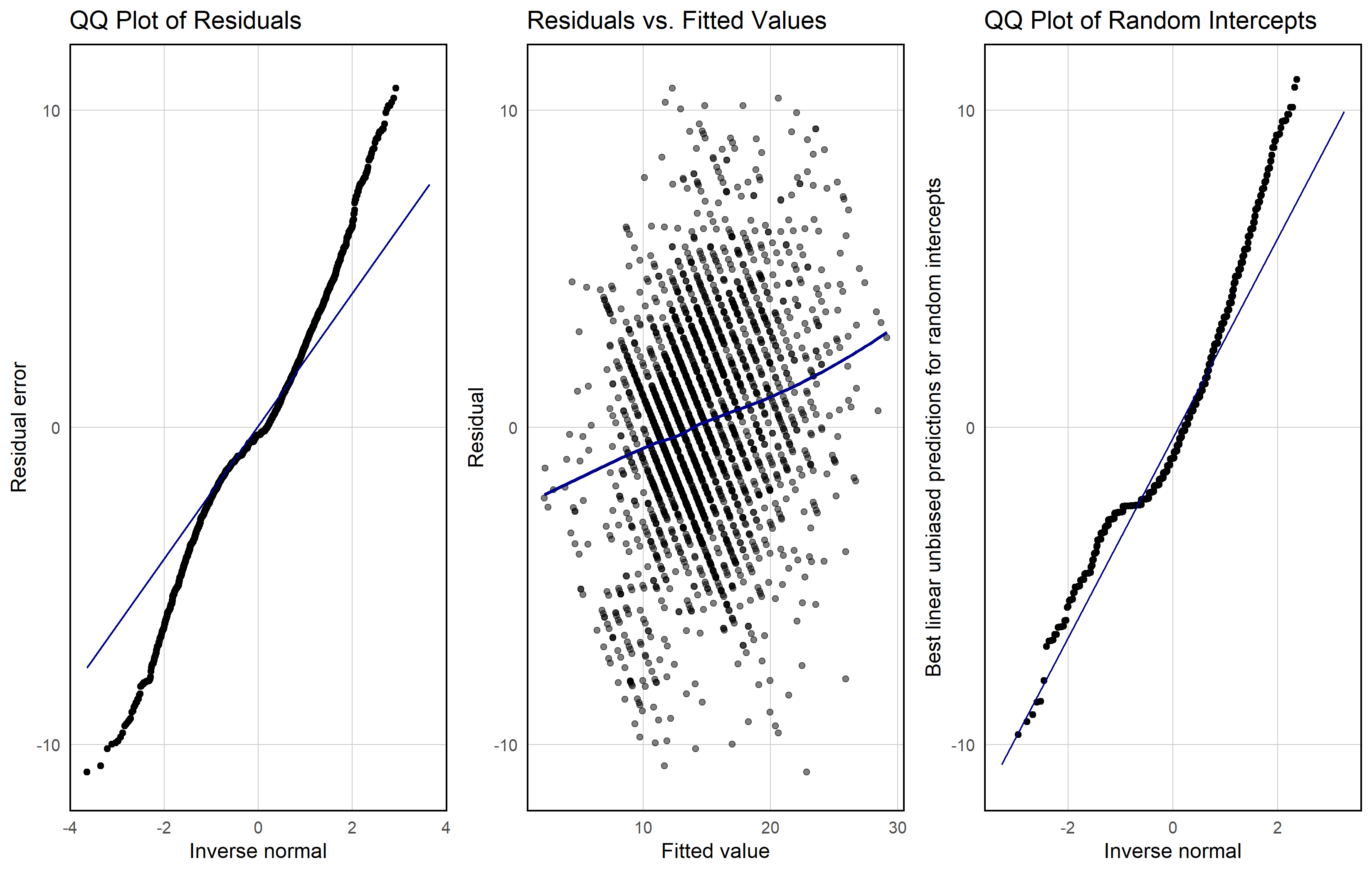


## Supplementary Figure 2. CFQ trajectory (Likert-style scoring system) model diagnostics.

*The initial model included CFQ total score (Likert-style scoring system) with a linear term for time since infection and a participant-level random intercept.*


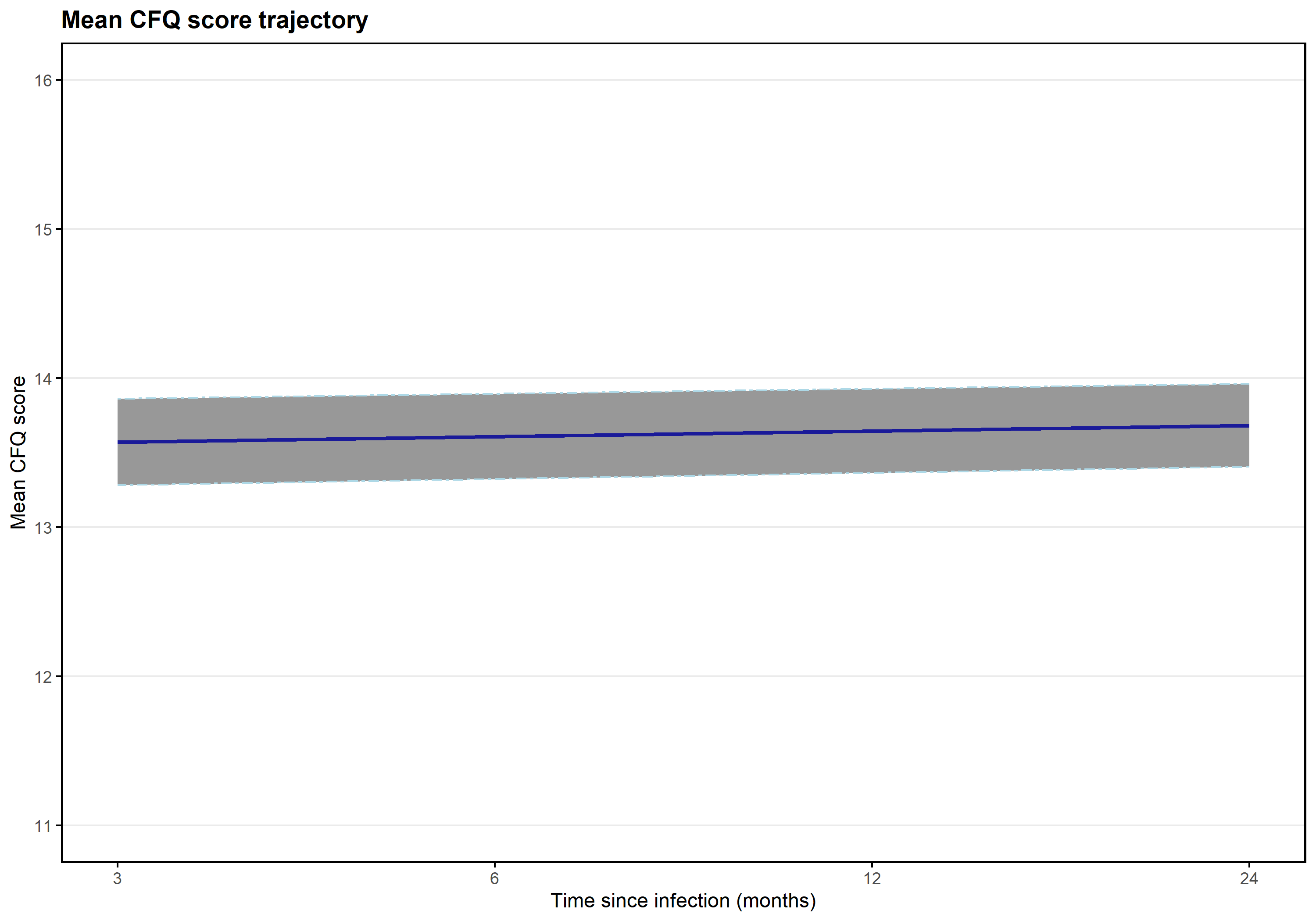


## Supplementary Figure 3. Trajectory of CFQ total score over time – scored according to the Likert-style scale scoring system (95% CI indicated via shading around trajectory).

*CFQ, Chalder Fatigue Scale.*

***
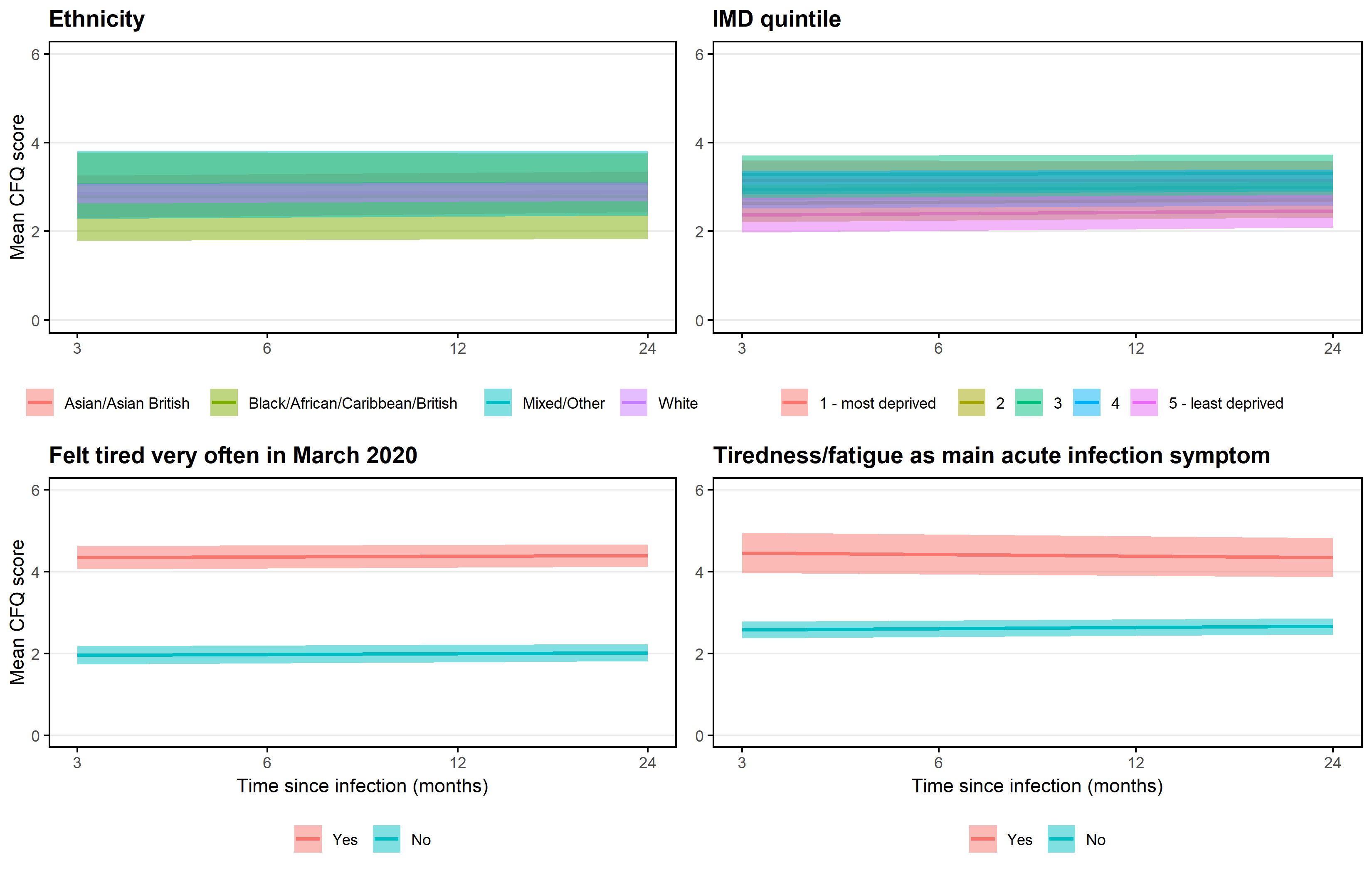
***

## Supplementary Figure 4. Trajectory of CFQ total score, by ethnicity, IMD quintile, feeling of tiredness very often in early in March 2020, and tiredness/fatigue as main acute infection symptom (95% CI indicated via shading around trajectory).

*CFQ, Chalder Fatigue Scale; IMD, index of multiple deprivation.*
